# Complete Genome Sequence of Halomonas hydrothermalis Strain Slthf2, a Halophilic Bacterium Isolated from a Deep-Sea Hydrothermal-Vent Environment

**DOI:** 10.1128/MRA.00294-20

**Published:** 2020-04-09

**Authors:** Naota Takeyama, Muyang Huang, Kensuke Sato, Josephine Galipon, Kazuharu Arakawa

**Affiliations:** a Institute for Advanced Biosciences, Keio University, Tsuruoka, Japan; b Faculty of Environment and Information Studies, Keio University, Fujisawa, Japan; c Systems Biology Program, Graduate School of Media and Governance, Keio University, Fujisawa, Japan; d Exploratory Research Center on Life and Living Systems, National Institutes of Natural Sciences, Myodaiji, Okazaki, Japan; Georgia Institute of Technology

## Abstract

Halomonas hydrothermalis strain Slthf2 is a Gram-negative bacterium isolated from low-temperature hydrothermal fluids in South Pacific Ocean vent fields located at 2,580-m depth. Here, we report the complete genome sequence of this strain, which has a genome size of 4.12 Mb, with a GC content of 53.2%.

## ANNOUNCEMENT

Halomonas hydrothermalis strain Slthf2 (ATCC BAA-800, CECT 5814, DSM 15725) is a Gram-negative, psychrotolerant, and moderately halophilic bacterium classified in the phylum *Proteobacteria*, order *Oceanospirillales*, and family *Halomonadaceae*. It was first isolated by Kaye et al. from low-temperature hydrothermal fluid at 2,580-m depth in the South Pacific Ocean near Easter Island (1). Some *Halomonas* strains produce polyhydroxybutyrate (PHB), which can be used as a substitute for petroleum plastics (2–4). In addition, a wide variety of carbon sources can be used for growth, including waste glycerol from the biodiesel manufacturing process, as well as PHB production (5). Therefore, here, we report the complete genome sequence of *H. hydrothermalis* strain Slthf2 to better understand the potential for industrial use of *Halomonas* species as PHB producers.

We picked one colony of *H. hydrothermalis* strain Slthf2 (obtained from J. Z. Kaye) and cultured it overnight at 37°C in SW10 culture solution, which had the following composition (% [wt/vol]): NaCl (8.1), MgCl_2_ (0.7), MgSO_4_ (0.96), CaCl_2_ (0.036), KCl (0.2), NaHCO_3_ (0.006), NaBr (0.0026), proteose peptone (Difco; 0.5), yeast extract (Difco; 1.0), glucose (0.1), and agar (1.5). The genomic DNA was extracted using Genomic-tip 20/G (Qiagen) according to the manufacturer’s protocol. The long-read sequence libraries were prepared using a rapid barcoding kit (SQK-RBK004; Oxford Nanopore Technologies) and were sequenced using the GridION device with a FLO-MIN106 flow cell (Oxford Nanopore Technologies). Illumina sequencing was performed for error correction by using a KAPA HyperPlus kit (Kapa Biosystems) for library preparation and a NextSeq 500 sequencer for sequencing using high-output mode and a run of 75 cycles (Illumina). Reads with at least 40,000 bp were used for the *de novo* assembly (71-fold coverage; 5,165 out of 266,015 sequenced reads) using Canu v1.8 (6), and the assembled single contig was manually circularized by eliminating an overlapping end. Contigs obtained from the assembly were further polished using Pilon v1.23 (7) with the short reads. The assembly completeness was assessed by BUSCO v1 (8) on the gVolante server (9), and the resulting genome sequence was functionally annotated using the DDBJ Fast Annotation and Submission Tool (DFAST) pipeline (10). The assembled genome consists of one circular chromosome of 4,120,823 bp having 53.2% GC content, including 3,761 coding sequences (CDSs), 60 tRNAs, and 18 rRNAs. All software programs were used with the default settings.

According to the annotation results, the genome of *H. hydrothermalis* strain Slthf2 encodes genes related to PHB production (LOCUS_04570), which is in agreement with previous reports (5). This information will be useful for revealing the production of PHB mediated by *H. hydrothermalis* and for contributing to the field of metabolic engineering.

### Data availability.

The chromosome sequence reported here was deposited in DDBJ under accession number AP022843, and the raw reads were deposited in the Sequence Read Archive (SRA) under BioProject accession number PRJNA606145.
